# Knowledge and attitudes of primary health care physicians and nurses with regard to population screening for colorectal cancer in Balearic Islands and Barcelona

**DOI:** 10.1186/1471-2407-10-500

**Published:** 2010-09-20

**Authors:** Maria Ramos, Magdalena Esteva, Jesús Almeda, Elena Cabeza, Diana Puente, Rosa Saladich, Albert Boada, Maria Llagostera

**Affiliations:** 1 Public Health Department, Balearic Islands Health Department, Spain; 2 Mallorca Primary Health Care Service, Balearic Island Health Service, Spain; 3 Costa de Ponent Primary Health Care Department, Catalonian Health Institut, Barcelona, Spain; 4 IDIAP-Jordi Gol, Barcelona, Spain; 5 CIBER, Epidemiology and Public Health (CIBERESP), Spain

## Abstract

**Background:**

Primary health care (PHC) professionals play a key role in population screening of colorectal cancer. The purposes of the study are: to assess knowledge and attitudes among PHC professionals with regard to colorectal cancer screening, as well as the factors that determine their support for such screening.

**Methods:**

Questionnaire-based survey of PHC physicians and nurses in the Balearic Islands and in a part of the metropolitan area of Barcelona.

**Results:**

We collected 1,219 questionnaires. About 84% of all professionals believe that screening for colorectal cancer by fecal occult blood test (FOBT) is effective. Around 68% would recommend to their clients a colorectal cancer screening program based on FOBT and colonoscopy. About 31% are reluctant or do not know. Professionals perceive the fear of undergoing a colonoscopy as the main obstacle in getting patients to participate, and the invasive nature of this test is the main reason behind their resistance to this program. The main barriers to support the screening program among PHC professionals are lack of knowledge (nurses) and lack of time (physicians). On multivariate analysis, the factors associated with reluctance to recommend colorectal cancer screening were: believing that FOBT has poor sensitivity and is complicated; that colonoscopy is an invasive procedure; that a lack of perceived benefit could discourage client participation; that only a minority of clients would participate; thinking that clients are fed up with screening tests and being unaware if they should be offered something to ensure their participation in the programme.

**Conclusions:**

Two in every three PHC professionals would support a population screening program for colorectal cancer screening. Factors associated with reluctance to recommend it were related with screening tests characteristics as sensitivity and complexity of FOBT, and also invasive feature of colonoscopy. Other factors were related with patients' believes.

## Background

Colorectal cancer is an important health problem in developed countries, both because of its high incidence and because it is accompanied by high mortality. In Spain, colorectal cancer ranks first among all cancers in terms of incidence and second in terms of mortality in both sexes together. Every year approximately 25,600 new cases are diagnosed [1] and in 2007 13,416 people died from the disease (INEbase). The annual adjusted incidence rates of colorectal cancer are under the average of the 25-member European Union (UE-25) in men, and especially in women. On the other hand, Spain's adjusted mortality rates are higher than the mean for the UE-25 in men, but lower in women [2].

There is hard evidence that colorectal cancer screening with fecal occult blood test (FOBT) and a colonoscopy on cases with positive FOBT, addressed to the population aged 50 to 74 years, effectively reduces colorectal cancer mortality and incidence [3], and clearly fulfills all the criteria of the World Health Organization for the development of a population screening program [4]. The European Union [5,6], together with the US Preventive Services Task Force [7] and the Canadian Task Force on Preventive Health Care [8] have been recommending it for years.

One crucial element for the success of population-based screening programs is the participation of a large percentage of the population (more than 50%) [9]. Thus, before a program of this type is implemented, how to ensure compliance is always of great concern. Additionally, it has been noted that individuals who refuse to participate are precisely those at highest risk of developing colorectal cancer [10].

Despite evidence of its effectiveness, organized population screening for colorectal cancer has only been implemented on a national scale in France, the United Kingdom, Finland and northen Italy [8,11]. In Spain, pilot tests have been carried out in certain regions, including Catalonia [12]. Experience has shown that PHC physicians play a key role, since people follow their advice when deciding whether to get screened or not [13,14]. Nonetheless, according to some studies PHC physicians have preconceived negative attitudes towards colon cancer screening programs that rely on FOBT [15,16]. The main reasons [17-21] are their skepticism regarding its effectiveness; concern over false positive and false negative results, i.e., the fear of "doing more harm than good"; doubts about patients' willingness to be tested in the absence of symptoms; lack of time and the added workload that could result from their participation in the program. At the same time, PHC nursing staff can also play an important role in colorectal cancer screening [22] because nurses usually devote more time than other professionals to health education activities [23].

This study is part of a more comprehensive study that aims to assess the knowledge and attitudes of PHC professionals as well as those of their patients with regard to population-based colorrectal cancer screening. The main objective of this first phase is to assess the knowledge and attitudes held by PHC physicians and nurses. Secondly, we want to identify the factors that determine the support afforded by these professionals to population screening for colorectal cancer.

## Methods

### Design

This is a cross-sectional descriptive multicentre study based on a survey conducted among adult care physicians and nurses in a primary health care setting in the Balearic Islands (1,014,405 inhabitants in 2007) and in the southern metropolitan area of Barcelona (1,275,679 inhabitants in the same year). Since the year 2000, a pilot program for population colorectal cancer screening based on FOBT and colonoscopy has been in place in the southern metropolitan area of Barcelona (115,867 inhabitants).

### Study population

In the Balearic Islands, all 1,001 professionals who were engaged in primary care at the time of the study (491 physicians and 510 nurses) were invited to participate. For the area of Barcelona, with 1,188 professionals (623 physicians and 565 nurses), a sample size was computed. The hypothesis was based on the assumption that 50% of professionals would support a population screening program. For a confidence level of 95%, and a precision of 5%, the required sample size was 297 physicians and 272 nurses. These were drawn from a convenience sample of health centres, including teaching and non-teaching centres and urban and rural centres.

### Data collection

The research team developed a questionnaire to be self-administered and based on a literature review [16,22,24]. In March 2008, we performed a pilot study in two health centres, one urban and the other rural, and collected 31 questionnaires that were then used to modify the wording or format of some of the questions. Between the months of April and June 2008, the definitive questionnaire, was completed by the health professionals in their own health center during one of their scheduled meetings. We also used some strategies to capture health professionals who were absent on the day the session was held.

### Variables

We explored the following variables: socio-demographic; professional; knowledge about colorectal cancer; knowledge about cancer screening; the performance of FOBT; attitudes towards implementing a colorectal cancer screening program based on FOBT and colonoscopy for cases with positive stools; perceived obstacles to client participation; reasons, from a professional standpoint, for reluctance to encourage clients to participate; barriers against enlisting the collaboration of PHC professionals and, finally, viewpoints on what to offer professionals to ensure their support to the program. For the knowledge variables and some of the attitudinal variables, the responses given were as follows: "I agree", "I disagree" and "I don't know". On the specific question of whether or not they would support a program of this type, the possible responses were: "Yes, enthusiastically", "Yes, with some reluctance", "No, absolutely not" and "I don't know". Regarding barriers and opportunities, the questionnaire offered an array of answers identified in previous studies. For each of the options, the professional could choose "Yes", "No" or "I don't know".

The study obtained the approval from the Primary Health Care Research Committee, the Balearic Islands Ethics Committee of Clinical Research and the IDIAP-Jordi Gol Ethics Committee. Informed consent to PHC professionals was not considered necessary.

### Analysis

We introduced the questionnaires into a database in Access using Teleform 4.0 for Windows.

In order to assess knowledge and attitudes held by physicians and nurses with regard to colorectal cancer screening, a descriptive analysis was performed. The sample structure was not taken into account in the analysis. We determined the frequencies of the categorical variables and assessed the normality of the continuous variables, whose mean and median we calculated. We carried out a bivariate analysis by type of professional using all variables. We used the chi squared test to compare hypotheses.

Secondly, to identify the factors related with the support afforded by PHC professionals to population screening for colorectal cancer, we carried out a bivariate analysis. Furthermore, a multivariate logistic regression analysis was performed using as dependent variable support or lack of support to the screening programme. Independent variables included were those showing a statistical significance of p < 0.05 in bivariate analysis. Backward logistic regression analysis was performed. Independent variables were excluded from the model when there was no statistical significative relationship with the dependent variable and when the estimated coefficients did not change markedly from the previous model with the variable. In addition, each new model was compared with the previous one through the likelihood ratio.

As software we used SPSS 13.0 for Windows.

## Results

We collected 1,219 questionnaires: 675 in the Balearic Islands (response rate: 67,4%) and 544 in Barcelona (95.6% of the anticipated sample). About 51.4% were physicians and 48.6% were nurses; 72.7% were women. The median age of the professionals was 46 years (interquartile range, IQR: 36-52). The median number of years spent working in PHC was 14 (IQR: 6-20). About 48.9% of the professionals were fixed staff, 28% were temporary staff, 12.5% were substitutes, 2.1% were residents and in 8.5% of the cases work status was unknown. About 52% worked in teaching health centres. The median number of consultations a day was 35 (IQR: 30-40) for physicians and 20 (IQR: 16-25) for nurses.

Interviewees' knowledge and beliefs regarding colorectal cancer, screening programs in general and colorectal cancer screening in particular are presented in Table 1. Professionals' attitudes regarding population screening for colorectal cancer based on testing FOBT and colonoscopy are presented in Table 2. It stands out that 68% of all professionals would recommend this programme to their clients and 31% are reluctant or do not know, with no differences noted between physicians and nurses. The majority (91.4% of nurses and 83.7% of physicians) of professionals were familiar with the procedure for FOBT, although only 74.8% of nurses and 60.6% of physicians explained to their clients how to perform the test.

**Table 1:** Knowledge and beliefs of primary care professionals about colorectal cancer, cancer screening programmes in general, and colorectal cancer screening in particular

Questions	Answers	Total (%)	Physicians (%)	Nurses (%)
Colorectal cancer is the most common cancer in both sexes together	I agree	58.5	69.0	47.1
	I disagree	17.9	17.8	18.1
	I don't know	23.6	13.2	34.8

Colorectal cancer is one of the three leading causes of death from cancer	I agree	77.6	87,5	66,9
	I disagree	8.7	7.3	10.2
	I don't know	13.7	5.2	22.9

About half of all people who have colorectal cancer are still alive 5 years after the diagnosis	I agree	69.6	80.1	58.3
	I disagree	8.1	8.3	8.0
	I don't know	22.3	11.6	33.7

The early diagnosis of colorectal cancer, before the onset of symptoms, is a prognostic factor	I agree	90.5	95.4	85.0
	I disagree	4.0	2.5	5.7
	I don't know	5.5	2.1	9.3

The rapid diagnosis of colorectal cancer, after the onset of symptoms, is a prognostic factor	I agree	80.9	84.3	76.0
	I disagree	11.5	12.5	10.4
	I don't know	7.6	3.2	12.8

The location of the colorectal cancer (colon or rectum) is a prognostic factor	I agree	70.2	71.0	69.3
	I disagree	10.7	14.3	6.0
	I don't know	19.1	14.8	24.6

Population screening programmes target asymptomatic subjects of specific age groups	I agree	87.1	93.4	80.2
	I disagree	5.0	3.7	6.3
	I don't know	8.0	2.9	13.5

The purpose of a population screening programme is to reduce the mortality rate	I agree	85.4	86.9	83.7
	I disagree	11.0	10.4	11.6
	I don't know	3.7	2.8	4.7

A screening programme's effectiveness depends on the % of the population that participates in it	I agree	70.0	76.5	63.0
	I disagree	14.4	13.2	15.7
	I don't know	15.6	10.3	21.3

Prostate cancer screening by testing for prostate-specific antigen (PSA) is...	Effective	76.4	64.8	88.8
	Ineffective	20.4	34.1	5.9
	I don't know	3.2	1.1	5.3

Breast cancer screening by means of mammography is...	Effective	98.3	99.2	97.4
	Ineffective	0.6	0.5	0.7
	I don't know	1.1	0.3	1.9

Lung cancer screening by means of CAT scans is...	Effective	78	67.2	89.5
	Ineffective	15.3	27.1	2.7
	I don't know	6.7	5.7	7.7

Screening for colorectal cancer by means of rectal examination is...	Effective	60.1	57.3	63
	Ineffective	30.1	39.5	20
	I don't know	9.9	3.3	17

Screening for colorectal cancer by testing for occult blood in stools (FOBT) is...	Effective	83.9	83.1	84.7
	Ineffective	11.3	15.1	7.1
	I don't know	4.9	1.8	8.2

Screening for colorectal cancer by means of colonoscopy is...	Effective	96.3	97.7	94.6
	Ineffective	1.0	1.6	0.3
	I don't know	2.8	0.6	5

The FOBT is too risky to be a screening test	I agree	4.3	3.0	5.7
	I disagree	83.3	92.4	72.7
	I don't know	12.5	4.6	21.5

Colonoscopy is too risky to be a screening test	I agree	33.4	36.6	29.8
	I disagree	48.9	54.2	42.9
	I don't know	17.6	9.2	27.4

**Table 2:** Primary care professionals' attitudes towards population screening for colorectal cancer

Questions	Answers	Total (%)	Physicians (%)	Nurses (%)
If a programme for population colorectal cancer screening based on FOBT and colonoscopy were implemented, would you recomend it?	Yes, enthusiastically	68.5	69.2	67.8
	Yes, with some reluctance	23.7	23.5	23.9
	I don't know	7.4	6.8	8.0
	No	0.4	0.5	0.3

How do you think the clients in your practice would react to a programme for population screening for colorectal cancer?	Almost everyone would participate	27.8	30.8	24.7
	About 50% would participate	36.2	40.2	31.8
	A minority would participate	14.5	15.1	13.8
	I don't know	21.5	13.8	29.7

For users, performing the FOBT is...	Easy	41.8	37.3	46.6
	Neither easy nor hard	40.8	42.4	39.0
	Complicated	14.2	16.9	11.2
	I don't know	3.3	3.4	3.3

What role do you think you could play in a colorectal cancer screening programme?*	General awareness-raising among clients about colorectal cancer and screening	92.2	90.8	93.6
	Individual counseling for reluctant clients	91.5	93.4	89.4
	Sending clients to the programme if they have not received the letter	77.6	77.9	77.2
	Capturing clients	74.3	74.1	74.5
	Signing letters inviting clients to join the programme	45.7	49.7	41.1

* professionals who agree with each statement, versus those who disagree + don't know

About 84.5% of the PHC physicians and 14.3% of the PHC nurses had recommended some type of screening test for colorectal cancer to their clients during the previous year. When we asked them to specify which clients, 26% of professionals who had recommended some type of screening test for colorectal cancer replied that it was those with family antecedents of colorectal cancer, and 23% that it was those with suspicious clinical symptoms, mainly anemia, rectal bleeding and changes in bowel habit.

The barriers perceived by PHC professionals with respect to population screening for colorectal cancer are shown in Table 3, together with their needs. Fear of having to undergo a colonoscopy is what professionals perceive to be the main obstacle to client participation. Similarly, the invasive nature of colonoscopy is the main reason for professionals' reluctance. The primary barriers in the way of professionals' support the screening program would be lack of knowledge for nurses and lack of time for physicians. What professionals need the most is training and information regarding the program.

**Table 3:** Barriers and needs surrounding population screening for colorectal cancer as perceived by primary care professionals*

Questions	Total (%)	Physicians (%)	Nurses (%)
¿What obstacles do you think we would encounter in trying to get your clients to participate?			
Fear of having to undergo a colonoscopy	71.1	73.3	68.6
Lack of knowledge about colorectal cancer	66.7	59.3	74.4
Fear of having a colorectal cancer found	60.2	60.1	60.3
The complicated nature of the procedure	41.9	49.3	33.7
Dislike or repulsion at having to handle stools	22.6	26.3	18.6
Perceiving no benefit	17.1	17.6	16.5
Lack of trust in the public health system	6.2	6.4	5.9

¿What would make you reluctant to encourage your clients to participate?			
Colonoscopy is an invasive procedure	60.9	56.4	65.8
The anxiety generated by false positive results	45.9	43.5	48.6
The fact that clients are fed up with screening tests	33.5	34.4	32.5
The false sense of security from false negative results	29.5	35.8	22.6

¿What are the main barriers to getting PHC professionals to support the screening program?			
Lack of time	88.9	93.6	83.9
Professional burnout	62.5	70.0	54.0
Difficulty in explaining this information to him/her	34.2	38.3	29.7
Lack of knowledge about screening programmes	33.9	26.3	42.2
Participation in other screening programmes	26.3	23.0	29.9
Lack of knowledge about colorectal cancer	24.8	15.4	35.0
Being disinterested in the matter	18.3	16.4	20.3

¿What do you think professionals should be offered to ensure their support?			
Training on colorectal cancer and screening	94.4	92.4	96.5
Detailed information about the programme	93.7	92.8	94.8
More time with each patient during patient visits	93.4	95.1	91.7
Regular feedback about the results	92.9	93.8	91.8
Collaboration in writing papers	74.1	76.6	71.4
Economic compensation	43.8	37.1	51.1
Nothing, since it is part of their work	22.0	23.0	20.9

*professionals who agree with each statement versus those who disagree + don't know

In bivariate analysis, several factors were associated with reluctance to support population screening for colorectal cancer, and they had to do with both knowledge and attitudes. We noted no differences by type of professional or area, an exception being the area in which the pilot study is being conducted, where the percentage of professionals who are reluctant to support a screening program for colorectal cancer is smaller than in the rest of the study area.

In the multivariate analysis (Table 4), reluctant professionals were found to be those who believe the FOBT has poor sensitivity and is difficult to perform; that colonoscopy is an invasive procedure; that not perceiving any benefit from screening could deter their clients from participating; that only a minority of their clients would take part in such a program; and, finally, resistance is felt by those who believe clients are fed up with screening tests and those who don't know if they should be offered something to ensure their support to the programme.

**Table 4:** Multivariate analysis of factors associated with being reluctant to support a population screening programme based on testing for occult blood in stools and colonoscopy*

Variable	Categories	p	OR	95% CI
Population screening programmes target asymptomatic subjects of specific age groups	I agree	0.041	1	
	I don't know	0.043	2.11	1.02-4.35
	I disagree	0.092	1.69	0.91-3.12
Test for occult blood in stools (FOBT) has poor sensitivity	No	0.008	1	
	I don't know	0.018	1.88	1.11-3.17
	Yes	0.004	1.71	1.18-2.48
Test for occult blood in stools (FOBT) has poor specificity	No	0.022	1	
	I don't know	0.467	0.80	0.45-1.44
	Yes	0.055	1.48	0.99-2.21
For users, performing the FOBT is...	Easy	0.002	1	
	Neither easy nor hard	0.009	1.62	1.12-2.32
	I don't know	0.008	3.26	1.36-7.84
	Complicated	0.002	2.06	1.29-3.28
How would people react to a population screening programme for colorectal cancer?	Almost everyone would participate	0.000	1	
	About 50% would participate	0.000	2.58	1.61-4.15
	A minority would participate	0.000	5.64	3.27-9.74
	I don't know	0.000	4.61	2.73-7.78
What barriers to client participation would we encounter?				
				
• Perceiving no benefit	I disagree	0.001	1	
	I don't know	0.002	1.99	1.28-3.07
	I agree	0.011	1.71	1.13-2.58
What would make you reluctant to enourage clients to participate?				
				
• Clients are fed up with screening programmes	I disagree	0.000	1	
	I don't know	0.555	1.17	0.69-1.99
	I agree	0.000	2.25	1.59-3.17
• Colonoscopy is an invasive procedure	I disagree	0.051	1	
	I don't know	0.781	1.13	0.47-2.71
	I agree	0.017	1.53	1.08-2.18
¿What do you think professionals should be offered to ensure their support to the screening program?				
• Nothing, since it is part of their work	I agree	0.113	1	
	I don't know	0.041	1.56	1.01-2.41
	I disagree	0.344	1.30	0.75-2.25

* Nagelkerke's R^2^: 0,257

## Discussion

We have explored the knowledge and attitudes of PHC professionals from two regions in Spain regarding population screening for colorectal cancer. We have obtained two kinds of responses: the ones associated to the professionals themselves, and the ones derived from their role as patient's advocates, that is, their opinion about how their patients would react towards a colorectal cancer screening programme. Patient's attitudes will be more thoroughly explored during the next phase of this study in which general population will be surveyed and interviewed.

In Spain, National Cancer Strategy [25] is promoting the development of population screening programs for colorectal cancer. Several regions are currently implementing them. All but one are centralized programs, in which PHC professionals play an insignificant role. The Basque country program, the only one pivoted around PHC professionals, is the one that until now has obtained the highest rates of participation, around 59% (data not published). In Cataluña, the pilot program for population colorectal cancer screening has used the guaiac FOBT test, obtaining low rates of participation in the first rounds, 17.5% in the first round and 22.3% in the second one [26]. Currently, the Cataluña Health Department has decided to move to the immunological FOBT test, used also in the other programs in Spain. In Balearic Island, such decision has not yet taken. Instead, the importance of pivoting the program around PHC professionals is clear for the Balearic Health Department, following the basque and the french models [27].

The strategy we followed yielded higher rates of PHC worker participation than we had expected in light of previous studies [16,28], which suggests that the subject is of interest to these workers. However, a 23% of professionals in Balearic Islands didn't answer the questionnaire. This fact can have produced a selection bias, since those who have not answered are probably the most skeptical. Another noteworthy limitation of the study is that we may have incurred in what may be referred to as "the public health expert's bias", since some of the questionnaire items were very technical and particularly difficult for nursing staff. However, when we designed the questionnaire we felt that anyone supporting a screening program has to be familiar with the health problem targeted by the program, the features of the test involved (sensitivity and specificity) and the programme expected benefits.

Most PHC physicians and nurses believe that colorectal cancer screening with FOBT is effective, although more than half of the physicians and most nurses also consider effective prostate cancer screening with prostate-specific antigen (PSA) and lung cancer screening with CAT scan. This casts doubt on their knowledge regarding the available evidence on the effectiveness of screening programs for different types of cancer. On the other hand, when we asked them if they had recommended some type of screening test for colorectal cancer to their clients over the previous year, most physicians said yes. Nonetheless, when asked to specify which clients, some professionals replied that it was those who had symptoms suggestive of colorectal cancer, which leads us to suspect that they do not fully understand what screening means. This is an important issue because lack of knowledge about the concept of population based screening programme was associated in the multivariant analysis to reluctance to support it.

Around 70% of primary care workers would enthusiastically support a population screening program for colorectal cancer based on a FOBT and a colonoscopy for cases with positive stools. This result is better than the one obtained in other countries such as Australia (50%) [24]. Another positive finding was that most professionals felt that their role in this type of program might be to raise general awareness among clients and counsel the ones showing reluctance. On the other hand, signing letters of invitation to join the program, a strategy that has effectively improved participation [29], was mentioned by less than half of all professionals, perhaps because Spanish physicians and nurses, and especially the former, dislike administrative tasks.

The main barriers expressed by PHC professsionals for supporting a colorectal cancer screening program were lack of knowledge among nurses and lack of time among physicians. Both aspects emerged in a previous qualitative study [21]. While improving the first factor is feasible, changing the second one is difficult in the short run. This, however, could be one more reason to leave nurses in charge of raising general awareness among users and counseling those harboring doubts, as the physicians themselves have suggested [30], especially since nurses appear to be more familiar than physicians with the procedure of FOBT, a factor that could also render them more willing to participate. In the United States, on the other hand, nurses' involvement in colorectal cancer screening is more geared towards performing sigmoidoscopies [31].

Health workers perceive the fear of having to undergo a colonoscopy as the most important barrier to client participation in a colorectal cancer screening program. This parallels their own reluctance, since they consider the procedure invasive and too hazardous for a screening test. The results of pilot studies carried out in Spain have shown that colonoscopy is acceptable to a high percentage − around 89% − of those who test positive for FOBT [26]. Despite this, health professionals and clients should be fully aware of the risks and complications of colonoscopy before subscribing to a program [32], and they should also be aware of its benefits, since this may encourage participation among professionals and, through their influence, among clients as well [33].

Besides from the invasive feature of colonoscopy, poor sensitivity and complicated procedure of FOBT were associated to reluctance to support a colorectal cancer screening program. FOBT poor sensitivity was also associated to supporting a colorectal cancer screening program in other studies [16,20], and also belief in FOBT test efficacy [24]. Clearly, immunological test improve guaiac test in both aspects [34]. Other factors associated with reluctance were related with PHC professional's believes about their patient's participation and tiredness with screening procedures. Both will be explored in depth in the next phase of the study.

## Conclusions

Two in every three PHC professionals would support a population screening program for colorectal cancer screening. Factors associated with reluctance to recommend it were related with screening test characteristics as sensitivity and complexity of FOBT and with invasive feature of colonoscopy. Other factors were related with patients' believes. Training programs will be needed before the population screening is launched, in order to maximize health professional's support.

## Competing interests

The authors declare that they have no competing interests.

## Authors' contributions

MR, ME and EC designed the study; MR and ML liderated the development of the study in Balearic Islands and Barcelona respectively; RS and AB coordinated the development of the study in their respective health areas; MR, ME, EC and JA performed the statistical analysis; MR, DP and JA draft the manuscript. ME, EC, RS, AB and ML review critically the draft. All authors read and approved the final manuscript.

## Pre-publication history

The pre-publication history for this paper can be accessed here:

http://www.biomedcentral.com/1471-2407/10/500/prepub
